# Comparative Analysis of Gut Microbiome, Metabolome and Meat Quality Among Chinese Steppe Red Cattle, Simmental Hybrid Cattle and Yanhuang Cattle

**DOI:** 10.3390/ani16162577

**Published:** 2026-08-18

**Authors:** Shiyu Guan, Ming Sun, Ning Xu, Yue Gu, Lihong Qin

**Affiliations:** 1 Jilin Academy of Agricultural Sciences (Northeast Agricultural Research Center of China), No. 1363, ShengTai Street, Changchun 130033, China; qqxiaocat0429@163.com (S.G.); 15590456660@163.com (M.S.); 13089512566@163.com (N.X.); 2 Jilin Science and Technology Innovation Center for Beef Cattle Breeding and Cultivation Technology, No. 1363, ShengTai Street, Changchun 130033, China; 3 College of Animal Science and Technology, Jilin Agricultural University, No. 2888, XinCheng Street, Changchun 130118, China; 4 Zhenlai County Hehe Animal Husbandry Development Co., Ltd., Baicheng 137300, China; chenchendk84@126.com

**Keywords:** Chinese steppe red cattle, rumen microbiota, metabolomics, meat quality, lipid metabolism

## Abstract

Breed is a major source of variation in beef quality, yet how differences in rumen microbial communities relate to metabolic profiles and meat quality across breeds remains unclear. This study compared growth performance, meat quality traits, rumen microbiota, and rumen metabolomic profiles of Chinese steppe red cattle (CR), Yanhuang cattle (YH), and Simmental hybrid cattle (SH). The results showed that SH cattle had better performance in some growth-related traits. In contrast, YH and CR cattle exhibited better meat quality, with higher marbling scores, lower drip loss and shear force, and higher levels of several unsaturated fatty acids. Significant differences in individual amino acid contents were also observed among breeds, and SH cattle showed higher levels of some amino acids. Microbial analysis showed that CR and YH cattle had higher rumen microbial richness and diversity than SH cattle, with differences in microbial community composition among breeds. A total of 2425 metabolites were annotated by metabolomic analysis. The differential metabolites were mainly involved in lipid metabolism, amino acid metabolism, carbon metabolism, and energy metabolism pathways. Correlation analysis further revealed relationships between specific rumen microorganisms and metabolites related to meat quality traits.

## Simple Summary

This study compares the differences in growth performance, meat quality, rumen microbiota, and metabolomic profiles among Chinese steppe red cattle, Simmental hybrid cattle, and Yanhuang cattle. The results revealed significant breed-specific variations in amino acid composition, fatty acid deposition, and rumen microbial composition. Yanhuang cattle and Chinese steppe red cattle exhibited higher rumen microbial diversity, greater enrichment of unsaturated fatty acids, and enhanced intramuscular fat deposition capacity, contributing to better overall meat quality than Simmental hybrid cattle and demonstrating their potential for high-quality beef production. Metabolic pathway analysis indicated that alterations in amino acid metabolism and lipid metabolism may represent important mechanisms underlying breed differences in meat quality traits. The rumen microbiota may regulate beef flavor, tenderness, and nutritional characteristics through interactions with host metabolic processes. In conclusion, this study reveals the differences in meat quality characteristics and the underlying molecular and microbial regulatory mechanisms among the three cattle breeds, providing an important theoretical basis for the utilization of high-quality beef cattle germplasm resources and the improvement of beef quality.

## 1. Introduction

As global demand for higher living standards rises, consumer preferences for meat have shifted from quantity toward quality [1]. Historically, livestock breeding programs have primarily focused on improving economically important production traits, such as growth rate and carcass yield, whereas meat quality traits received relatively limited attention [2,3]. In recent years, consumer demand for premium beef with superior eating quality and nutritional value has increased substantially [4]. However, beef cattle improvement in China has increasingly relied on crossbreeding with a few specialized beef breeds, particularly Simmental cattle. Although these strategies improve growth performance and carcass traits, they may reduce the use of indigenous cattle resources and their unique meat quality traits [5,6]. To satisfy the increasing consumer demand for premium beef, improving meat quality through the selection and breeding of genetically superior beef cattle has become a major objective of modern breeding programs [2]. Because meat quality is strongly influenced by breed, evaluating the meat quality characteristics of different beef cattle breeds is essential for genetic improvement and the development of high-quality beef production [7,8]. Consequently, comparative studies of representative cattle breeds provide valuable information for identifying superior genetic resources and establishing a scientific basis for the genetic improvement and sustainable utilization of beef cattle [9]. Therefore, there is an urgent need to meet rising demand for premium beef while accelerating breeding programs to improve native cattle lines. In northeast China, Chinese steppe red, Simmental hybrids, and Yanhuang cattle stand out for traits relevant to this goal: strong cold tolerance, high disease resistance, rapid growth, and excellent meat quality.

The rumen microbiota is a highly complex and diverse microbial ecosystem. Microorganisms in the rumen maintain a dynamic equilibrium through cooperative and competitive interactions [10,11,12]. This microbiota is intricately linked to several physiological functions of the host, including metabolism, immune regulation, growth and development, and production performance [13,14,15,16]. In contrast to humans, the ruminant rumen can acquire and absorb certain nutrients that the host cannot digest [17]. The gastrointestinal microbiota of ruminants plays a pivotal role in regulating nutrient utilization, animal health, production performance, and meat quality [18]. The composition and concentration of fatty acids in ruminants are primarily determined by dietary nutrition, rumen microbial metabolism, and the regulation of fat metabolism. In ruminants, dietary lipids undergo extensive microbial lipolysis and biohydrogenation in the rumen. These microbial processes determine the profile of fatty acids available for intestinal absorption and consequently influence the fatty acid composition of muscle tissue [19]. In ruminants, the rumen microbial community can independently regulate the host’s metabolism, and alterations in its community composition can directly influence the host’s phenotype [20]. Research indicates that by modulating the rumen microbial community of ruminants, it is possible to alter the composition of meat amino acids and fatty acids, which in turn can affect muscle metabolism [21]. Wang et al. found that restructuring the rumen bacterial community influences the amino acid and fatty acid composition of lamb meat, as well as related biological pathways [21]. Kim et al. reported a strong correlation between increased microbial richness and both higher marbling scores and greater lipid content in the longus dorsi muscle. Specific taxa associated with marbling in Korean beef cattle, including Verrucomicrobia, were identified, suggesting that elevated intramuscular fat may reflect expanded ruminal bacterial diversity [22]. Furthermore, the majority of the microbiota related to intramuscular and subcutaneous fat deposition do not overlap, indicating that the end products of the gastrointestinal microbiota may influence fat accumulation through a distinct adipogenic pathway in the host animal [23].

Metabolomics, which has emerged after genomics, transcriptomics, and proteomics, extends the latter two by providing a more direct and accurate measure of an organism’s physiological state [24]. Metabolites are central to cellular biochemistry, functioning as substrates, intermediates, or final products of metabolic processes [25]. Studies have shown that metabolites affect meat texture, flavor, and color stability [26]. In a study by Zhang et al., metabolomics was used to show that traditionally grazing goats had poorer meat quality than caged goats because of reduced unsaturated fatty acid biosynthesis [27]. Therefore, comparing rumen metabolism across Chinese steppe red cattle, Simmental hybrid cattle, and Yanhuang cattle using metabolomics can provide novel insights for this study.

Differences in growth performance and meat quality among Chinese steppe red cattle, Yanhuang cattle, and Simmental hybrid cattle may be associated with the specific composition, structure, and metabolic functions of the rumen microbiota. In this study, growth and meat quality indicators were evaluated in 18-month-old CR, SH, and YH individuals. The structure, functions, and metabolic pathways of the rumen microbiota were identified using microbiomics and metabolomics technologies. Correlation analysis was further applied to assess the relationships between these indicators and meat quality traits, aiming to provide potential novel biomarkers for improving beef quality.

## 2. Materials and Methods

### 2.1. Animals, Housing, and Feeding

In this study, 18 cattle aged 18 months were selected, including Chinese steppe red cattle (CR, *n* = 6), Simmental hybrid cattle (Simmental × local Yellow cattle; SH, *n* = 6), and Yanhuang cattle (YH, *n* = 6). All experimental animals were obtained from a beef cattle breeding farm in Jilin Province at approximately 5 months of age, with initial body weights ranging from 170 to 180 kg. The cattle were maintained under identical feeding conditions, standardized management practices, and the same dietary formulation throughout the experimental period. The ingredients and nutritional levels of the experimental diets are shown in Table 1.

### 2.2. Sample Collection

Before slaughter, all cattle were fasted for 24 h, with free access to drinking water until 12 h before slaughter. Following the fasting period, live body weight was recorded. Body conformation traits, including body length, body height, hip height, chest girth, chest width, abdominal circumference, and chest depth, were measured for each animal.

Approximately 6 h before slaughter, rumen fluid samples were collected from each animal using a sterile rumen sampler while wearing sterile gloves. The rumen fluid was immediately transferred into sterile cryogenic tubes, snap-frozen in liquid nitrogen, and stored at −80 °C until microbial diversity and metabolomic analyses.

All cattle were slaughtered individually on the same day using a commercial automatic slaughter line under standard commercial procedures. After slaughter, carcass weight, net meat weight, and bone weight were recorded.

Longissimus thoracis muscle samples were collected from the left side of each carcass between the 12th and 13th ribs immediately after slaughter. Visible subcutaneous fat and connective tissue were carefully removed. One portion of the muscle was used for the determination of meat quality traits, including pH, eye muscle area, backfat thickness, marbling score, drip loss, and shear force. The remaining muscle samples were divided into appropriate portions, immediately snap-frozen in liquid nitrogen, and stored at −80 °C for subsequent analyses of fatty acid composition, amino acid composition, and other biochemical analyses.

### 2.3. Meat Quality Traits Detection

At 24 h after slaughter, the left longissimus dorsi muscle between the 12th and 13th ribs was exposed, and the complete cross-sectional outline of the muscle was traced on transparent tracing paper. The traced outline was scanned, and the eye muscle area was calculated based on the enclosed area according to the method described previously [28].

Backfat thickness was measured at the subcutaneous fat layer between the 12th and 13th ribs using a Vernier caliper [29]. The pH value of the longissimus dorsi muscle was determined using a portable penetration pH meter equipped with a glass electrode. Before measurement, the pH meter was calibrated using standard buffer solutions (pH 4.00 and 7.00). The electrode was inserted into the muscle at multiple locations while avoiding visible fat and connective tissue. The mean value of the measurements was used for statistical analysis [30]. Marbling was evaluated on the cross-section of the eye muscle at the 12th–13th thoracic intercostal space. According to the distribution, content, and uniformity of intramuscular fat, the marbling grade was assessed by comparison with the standard marbling pattern chart [31]. For drip loss measurement, three small samples of longissimus dorsi muscle were collected and weighed, respectively, as initial weight (W_1_). Each sample was suspended in a plastic cup, and the cup mouth was sealed with plastic wrap. After storage at 4 °C for 24 h, the samples were dried with filter paper and weighed again as final weight (W_2_). The calculation formula was as follows: Drip loss (%) = (W_1_ − W_2_)/W_1_ × 100% [32]. Shear force was measured using a C-LM tenderness meter (Shanghai Precision Scientific Instrument Co., Ltd., Shanghai, China). The muscle shear force was vacuum-packaged and cooked in a water bath until the internal temperature reached 70 °C. After cooling to room temperature, cylindrical cores (1.27 cm in diameter) were removed parallel to the muscle fiber orientation. Each core was sheared once perpendicular to the muscle fiber direction, and at least six cores were measured for each sample. The average shear force value was used for statistical analysis [33]. Amino acids were determined by an automatic amino acid analyzer, and the detailed determination procedure referred to the published literature [34].

The quantification of 37 fatty acids in beef was conducted using gas chromatography-tandem mass spectrometry (GC-MS/MS) following the group standard T/JCBD 002-2025 [35].

All experimental sample data were averaged for subsequent statistical analysis.

### 2.4. Microbiome Analysis of Rumen Microbiota

Total genomic DNA was extracted from bovine rumen fluid samples using the TGuide S96 Magnetic Stool DNA Kit (Tiangen Biotech (Beijing) Co., Ltd., Beijing, China) according to the manufacturer’s instructions. DNA concentration and quality were quantified using a NanoDrop 2000 UV-Vis spectrophotometer (Thermo Scientific, Wilmington, NC, USA). The primer sequences were as follows: 27F (5′-AGRGTTTGATYNTGGCTCAG-3′) and 1492R (5′-TASGGHTACCTTGTTASGACTT-3′). The pooled PCR products were subjected to damage repair, end repair, and adapter ligation using the SMRTbell Template Prep Kit (Pacific Biosciences, Menlo Park, CA, USA). The reaction procedures were performed on a PCR instrument. Subsequently, the library was purified and recovered with AMPure PB magnetic (Brea, CA, USA) beads to obtain the sequencing library. The quality of each library was evaluated by concentration quantification using a Qubit fluorometer (Carlsbad, CA, USA) and fragment size detection using an Agilent 2100 Bioanalyzer (Agilent Technologies, Santa Clara, CA, USA). All libraries were sequenced on the PacBio sequencing platform.

The_Q1 raw reads generated from sequencing were filtered and demultiplexed using SMRT Link software (version 8.0) with minPasses ≥ 5 and minPredictedAccuracy ≥ 0.9 in order to obtain circular consensus sequencing (CCS). Subsequently, Cutadapt v2.7 was used to recognize and remove primer sequences, followed by sequence length filtering [36]. The UCHIME algorithm (v8.1) was used to detect and remove chimera sequences to obtain the clean reads [37]. Sequences with similarity > 97% were clustered into the same operational taxonomic unit (OTU) by USEARCH (v10.0) [38,39,40,41]. Taxonomy annotation of the OTUs was performed based on the Naive Bayes classifier in QIIME2 using the SILVA database (release 138.1) with a confidence threshold of 70% [42,43]. Alpha diversity indices were calculated using mothur software (v1.48.0). Principal coordinate analysis (PCoA) based on the Bray–Curtis distance algorithm was performed to examine the similarity of microbial community structure among samples. To identify core differential taxa with higher effect sizes and well-defined biological significance, while reducing false positive interference from multiple comparisons, the linear discriminant analysis (LDA) score threshold was set to 4 in the present study. Only taxa with an LDA score > 4 and *p* < 0.05 were defined as significantly differential taxa among groups [44,45].

### 2.5. Metabolomic Analysis

For each rumen fluid sample, 100 μL was transferred into a 2 mL centrifuge tube with 500 μL of extraction solution (methanol/acetonitrile = 1:1, *v*/*v*) at 4 °C. The mixture was ultrasonicated for 10 min and then centrifuged to collect the supernatant. Chromatographic separation was performed on a Thermo Ultimate 3000 system (Thermo Scientific, Wilmington, NC, USA) equipped with an Acuity UPLC^®^ HSS T3 column (150 mm × 2.1 mm, 1.8 μm, Waters, Milford, MA, USA), followed by mass spectrometric detection. The MS1 and MS2 spectral data were acquired using a Waters Xevo G2-XS QTof high-resolution mass spectrometer (Waters, Milford, MA, USA).

The parameters of the ESI ion source were set as follows: Capillary voltage: 2000 V in positive ion mode and −1500 V in negative ion mode; Cone voltage: 30 V; Ion source temperature: 150 °C; Desolvation gas temperature:500 °C; Cone gas flow rate: 50 L/h; Desolvation gas flow rate: 800 L/h [46].

### 2.6. Statistical Analysis

The Kruskal–Wallis test was used to analyze the intergroup variables of the microbiome and metabolome. One-way analysis of variance (One-way ANOVA) was used for statistical analysis of growth performance and meat quality-related indicators. Data analysis was performed with GraphPad Prism 9.0.0 (GraphPad Software, San Diego, CA, USA). After differential bacterial taxa were screened via microbial diversity analysis, correlation analysis was conducted between these differential taxa and differential metabolites, as well as growth and meat quality indicators. Correlation calculations and visualization were implemented in R software (v4.3.2), with Spearman correlation analyses and significance testing performed using built-in functions, and correlation plots generated via the ggplot2 (v3.4.4) and corrplot (v0.94) packages.

## 3. Results

### 3.1. Determination of Growth Performance

The measurements of body length, body height, hip cross height, chest girth, and abdominal girth showed that the hip cross height of CR was significantly lower than that of SH and YH, while there were no significant differences in body length, body height, chest girth, and abdominal girth among the groups (*p* < 0.05; Table 2).

### 3.2. Meat Quality Trait Measurement

Three breeds of cattle were slaughtered, and meat quality-related traits were determined after slaughter. The results showed that there were no significant differences in live weight, carcass weight, net meat weight, bone weight, dressing percentage, and net meat percentage among the three breeds (*p* > 0.05; Table 2). The eye muscle area and backfat thickness of SH were significantly higher than those of YH and CR; no significant difference was observed in pH. The marbling score of CR and YH was significantly higher than that of SH. The drip loss rate and shear force of SH were significantly higher than those of YH and CR (*p* < 0.05; Table 2). This indicates that the meat quality of Chinese steppe red cattle and Yanhuang cattle was superior to that of Simmental hybrid cattle.

### 3.3. Amino Acid and Fatty Acid Composition in CR, SH, and YH Groups

The amino acid and fatty acid contents of meat were determined. The results showed that the contents of Asp, Arg, Phe, Ile, Leu, and Lys in SH were significantly higher than those in YH and CR. There were no significant differences in Glu, Ser, and Tyr among the three cattle breeds. The contents of His, Gly, Ala, Val, and Pro in SH and CR were significantly higher than those in YH, while the contents of Thr and Met in YH were significantly higher than those in CR and SH (*p* < 0.05; Table 2).

In addition, targeted fatty acid metabolomics analysis was performed to investigate the fatty acid composition of meat from the three cattle breeds. The results revealed significant differences in the contents of seven fatty acids among the three breeds. The levels of MA, PA, POA, SA, OA, LA, and ALA in YH and CR were significantly higher than those in SH (*p* < 0.05; Table 2). These components may play an important role in the formation of meat flavor.

### 3.4. Study on Microbial Flora in Rumen Fluid

Raw circular consensus sequencing (CCS) reads generated by the PacBio platform were subjected to rigorous filtering to remove low-quality reads, short fragments, and chimeric sequences. Finally, 1,017,558 valid sequences were obtained for subsequent OTU clustering at a 97% sequence similarity threshold.

**Figure 1:**
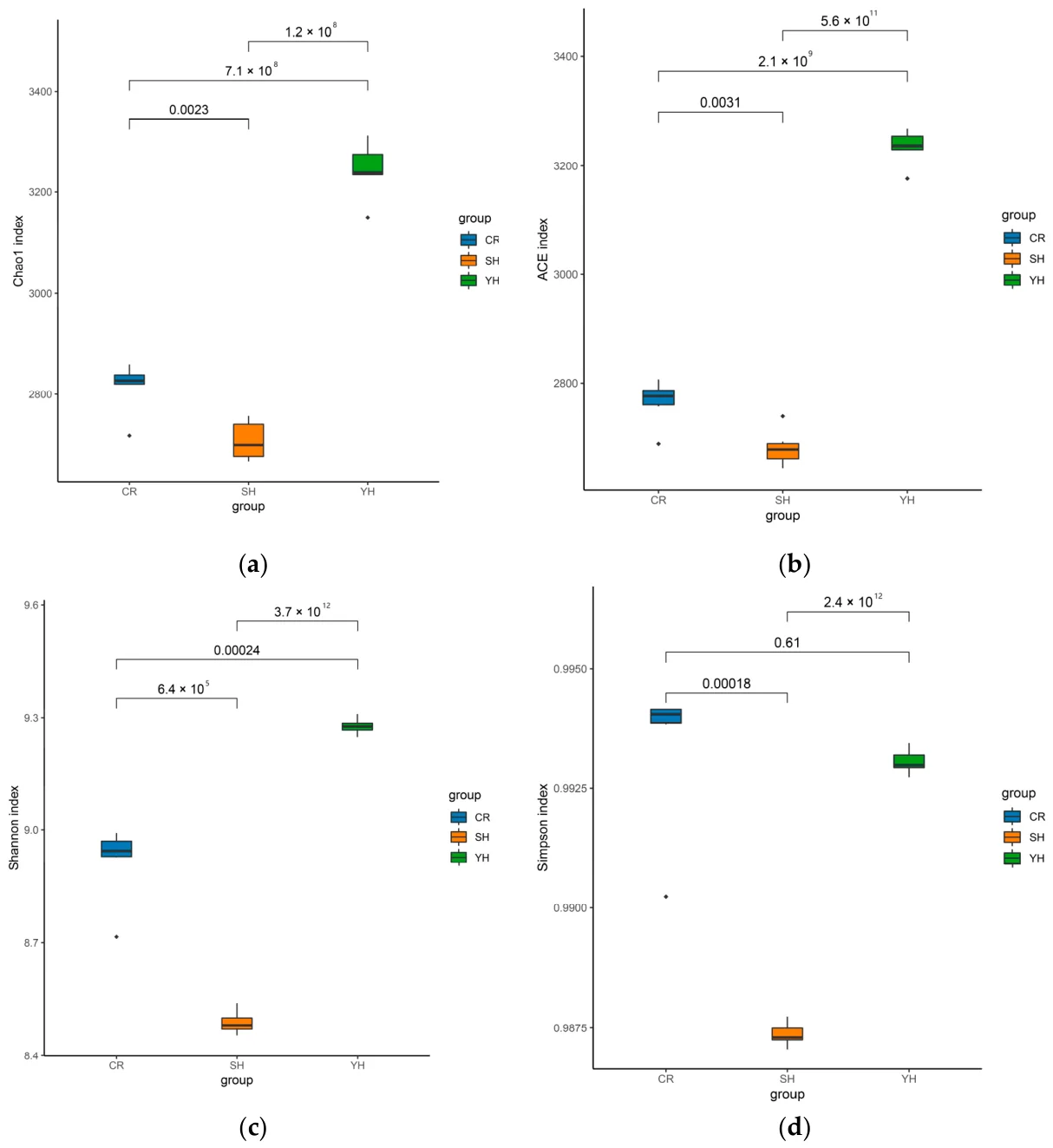
Comparison of alpha-diversity in the rumen microbiota between CR, SH, and YH. (*n* = 6 in each breed). (**a**) The ACE estimator; (**b**) The chao1 estimator; (**c**) Shannon diversity index; (**d**) Simpson diversity index; Values were expressed as means ± SEM.

**Figure 2:**
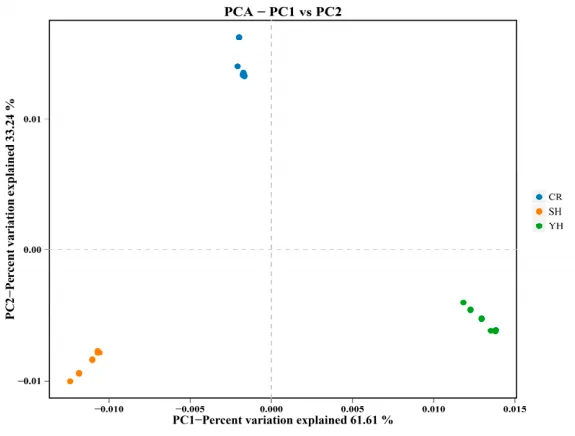
Comparison of beta-diversity in the rumen fluid microbiota between CR, SH, and YH. (*n* = 6 in each breed).

The differences in alpha diversity indices (Simpson, Chao1, ACE, and Shannon indices) and beta diversity of rumen fluid microbial communities among the three cattle breeds are shown in Figure 1a–d. The Chao1, ACE, Shannon, and Simpson indices in the SH group were significantly lower than those in the CR and YH groups (*p* < 0.05). As shown in Figure 2, principal coordinate analysis (PCoA) was used to evaluate beta diversity among different samples, and the results revealed obvious independent distribution differences among CR, SH, and YH groups (ANOSIM test, *p* < 0.05).

**Figure 3:**
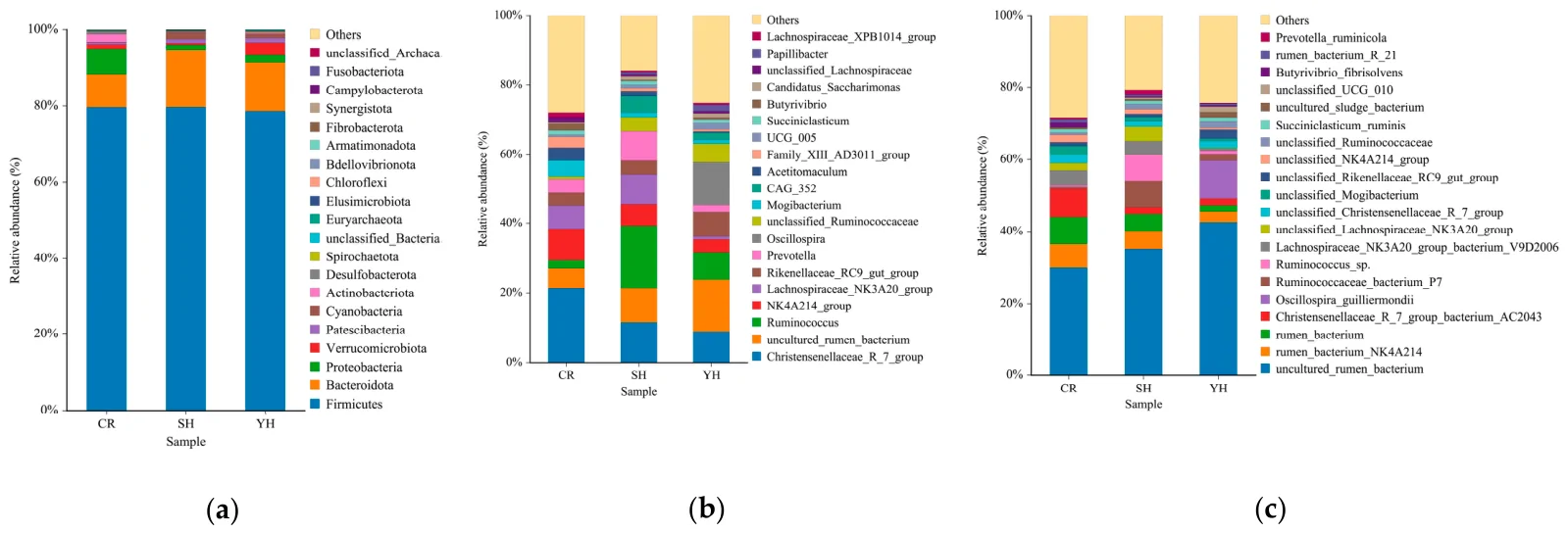
Microbial composition of rumen fluid between CR, SH, and YH. (**a**) The differential Phylum at different relative abundances. (**b**) The differential Genus at different relative abundances. (**c**) The differential Species at different relative abundances.

As shown in Figure 3a, at the phylum level, the dominant rumen bacterial phyla included Firmicutes (CR: 79.66%, SH: 79.69%, YH: 78.67%), Bacteroidota (CR: 8.63%, SH: 15.11%, YH: 12.68%), and Proteobacteria (CR: 6.78%, SH: 1.27%, YH: 1.97%). As shown in Figure 3b, at the genus level, the predominant rumen genera were *Christensenellaceae_R_7_group* (CR: 21.42%, SH: 11.46%, YH: 12.68%), uncultured_rumen_bacterium (CR: 5.84%, SH: 9.92%, YH: 15.04%), and *Ruminococcus* (CR: 2.29%, SH: 17.88%, YH: 7.83%). As shown in Figure 3c, at the species level, the dominant rumen species consisted of *Christensenellaceae_R_7_group* (CR: 21.42%, SH: 11.46%, YH: 12.68%), uncultured_rumen_bacterium (CR: 5.84%, SH: 9.92%, YH: 15.04%), and *Ruminococcus* (CR: 2.29%, SH: 17.88%, YH: 7.83%).

**Figure 4:**
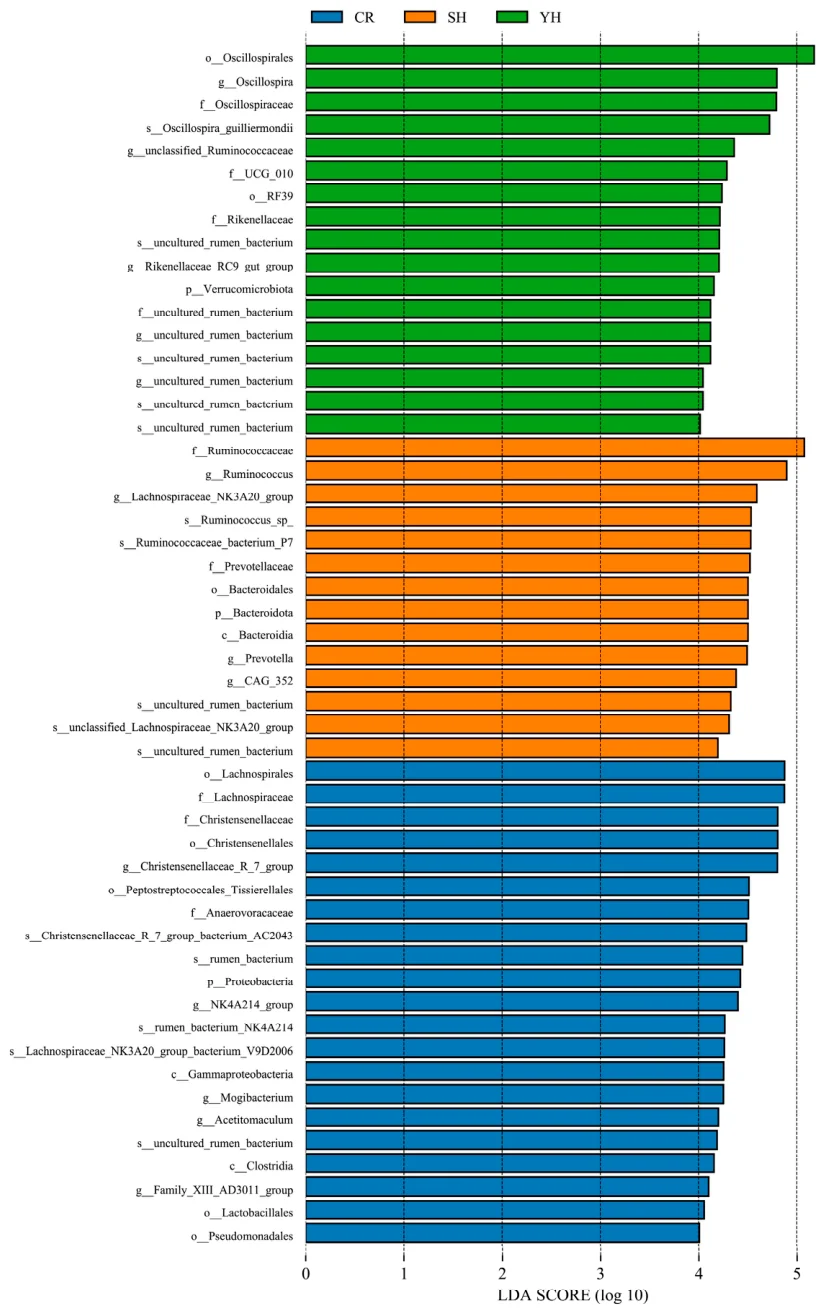
LEfSe analysis in the rumen fluid microbiota between CR, SH, and YH. (*n* = 6 in each breed). LEfSe, Linear discriminant analysis Effect Size.

Using LEfSe analysis (LDA score > 4), we identified biomarker taxa with significant intergroup differences from the phylum to species levels among SH, CR, and YH groups (Figure 4). At the phylum level, Proteobacteria was significantly enriched in the CR group, whereas Bacteroidota was significantly enriched in the SH group, and Verrucomicrobiota was significantly enriched in the YH group. At the genus level, 5 bacterial genera were significantly enriched in the CR group, 4 bacterial genera were significantly enriched in the SH group, and 5 bacterial genera were significantly enriched in the YH group. At the species level, 5 bacterial species were significantly enriched in the CR group, 5 bacterial species showed significant differences in the SH group, and 5 bacterial species showed significant differences in the YH group (Figure 4).

### 3.5. Metabolomic Study of Rumen Fluid

**Figure 5:**
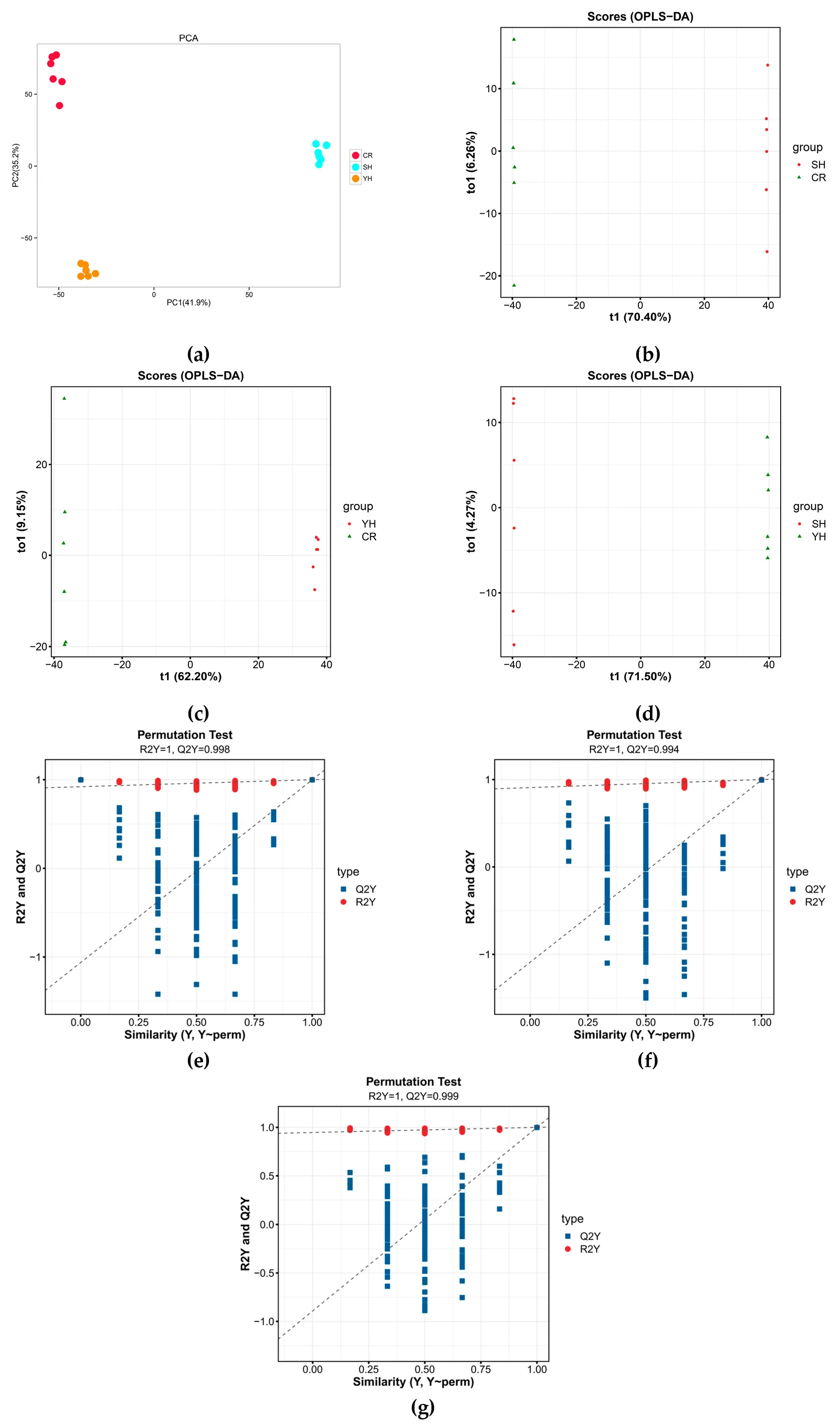
Metabolomic analysis of rumen fluid in CR, SH, and YH breeds. (**a**) Principal component analysis (PCA); (**b**) Orthogonal partial least squares discriminant analysis (OPLS-DA) score plot of rumen fluid metabolome between CR and SH; (**c**) OPLS-DA score plot of rumen fluid metabolome between CR and YH; (**d**) OPLS-DA score plot of rumen fluid metabolome between SH and YH; (**e**) OPLS-DA permutation test plot of rumen fluid metabolome between CR and SH; (**f**) OPLS-DA permutation test plot of rumen fluid metabolome between CR and YH; (**g**) OPLS-DA permutation test plot of rumen fluid metabolome between SH and YH.

Untargeted metabolomic qualitative and quantitative analysis was performed on 18 rumen fluid samples, and a total of 2425 metabolites were identified and annotated. Principal component analysis (PCA) revealed high metabolic variation among different groups and low variation within each group (Figure 5a). In addition, orthogonal partial least squares discriminant analysis (OPLS-DA) showed obvious distinguishable separation among all groups (Figure 5b–d). The robustness of the OPLS-DA models was further confirmed by permutation tests, indicating that the observed separation was not attributable to model overfitting (Figure 5e–g).

**Figure 6:**
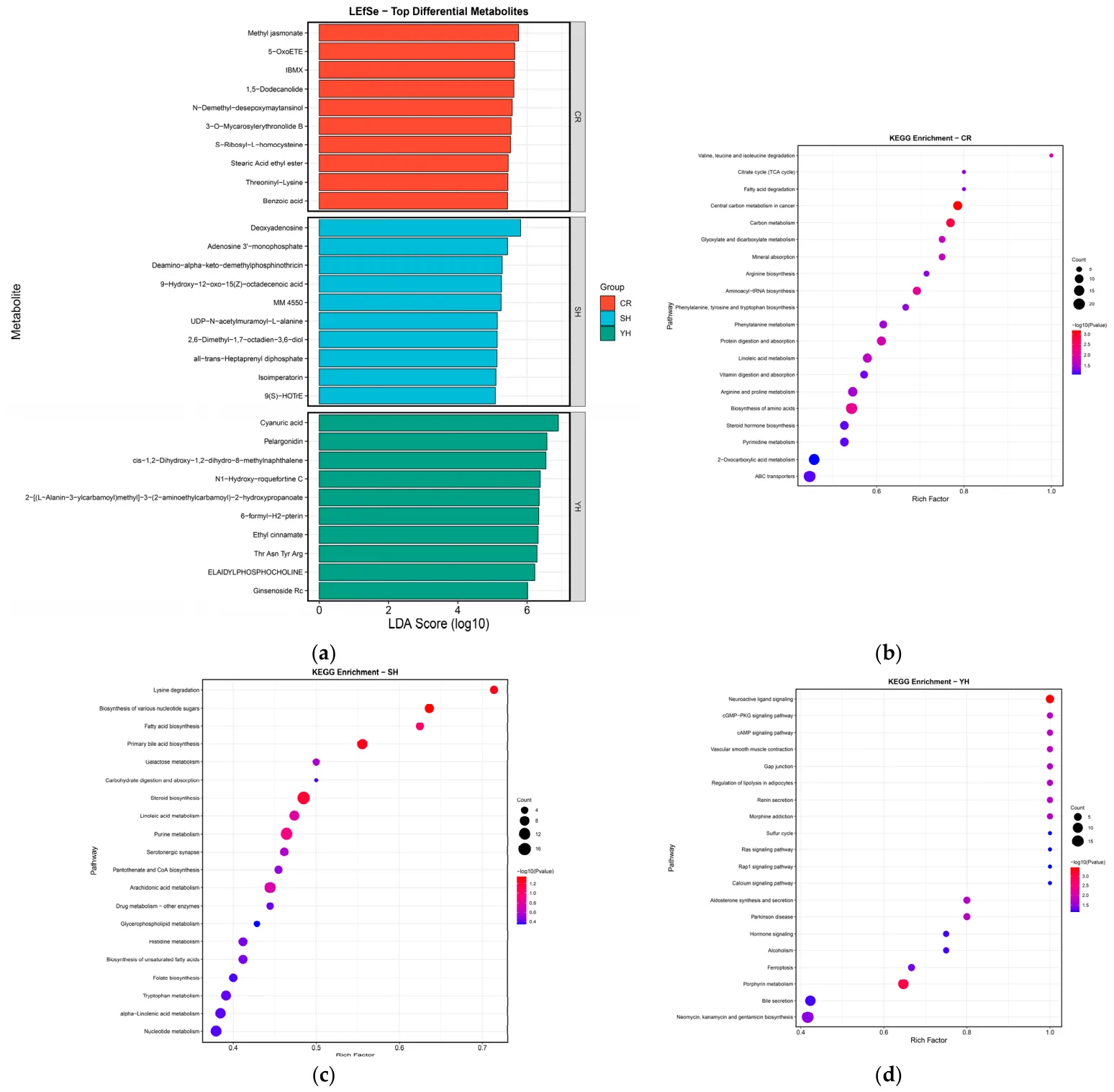
Differential Metabolite Screening and Pathway Enrichment. (**a**) LEfSe analysis of rumen fluid metabolites between CR, SH, and YH. (*n* = 6 in each breed). LEfSe, Linear discriminant analysis Effect Size. (**b**) KEGG pathway enrichment analysis was performed on differential metabolites enriched in the CR. (**c**) KEGG pathway enrichment analysis was performed on differential metabolites enriched in the SH. (**d**) KEGG pathway enrichment analysis was performed on differential metabolites enriched in the YH.

As shown in Figure 6a, a total of 83 significantly differential metabolites were identified among the CR, SH, and YH groups via LEfSe analysis (LDA > 5). To intuitively visualize the signature differential metabolites of each group and streamline the visualization figures, only the top 10 differential metabolites ranked by LDA score from each group were selected to generate bar charts for comparing characteristic metabolic profiles across groups. Detailed information on all 83 differential metabolites is provided in Supplementary Table S1.

Thirty-four metabolites were significantly enriched in the CR group. Subsequent KEGG enrichment analysis of these CR-enriched metabolites revealed significant enrichment in pathways including branched-chain amino acid degradation, tricarboxylic acid (TCA) cycle, carbon metabolism, amino acid biosynthesis, and nutrient absorption and transport, alongside lipid metabolic pathways such as linoleic acid metabolism and steroid biosynthesis (Figure 6b).

Eleven metabolites were significantly enriched in the SH group. KEGG enrichment of these metabolites demonstrated dominant pathways of fatty acid biosynthesis, polyunsaturated fatty acid metabolism, amino acid degradation, as well as carbohydrate and purine nucleotide metabolism, with remarkable enrichment of multiple lipid synthesis and polyunsaturated fatty acid metabolic pathways (Figure 6c).

Furthermore, thirty-eight metabolites were significantly enriched in the YH group. KEGG enrichment analysis of the YH-specific enriched metabolites identified prominent enrichment in intracellular signaling pathways such as cAMP/cGMP-PKG, Ras/Rap1 and calcium signaling, together with pathways related to hormone secretion, lipolysis, vascular smooth muscle contraction, neural regulation, and ferroptosis (Figure 6d).

**Figure 7:**
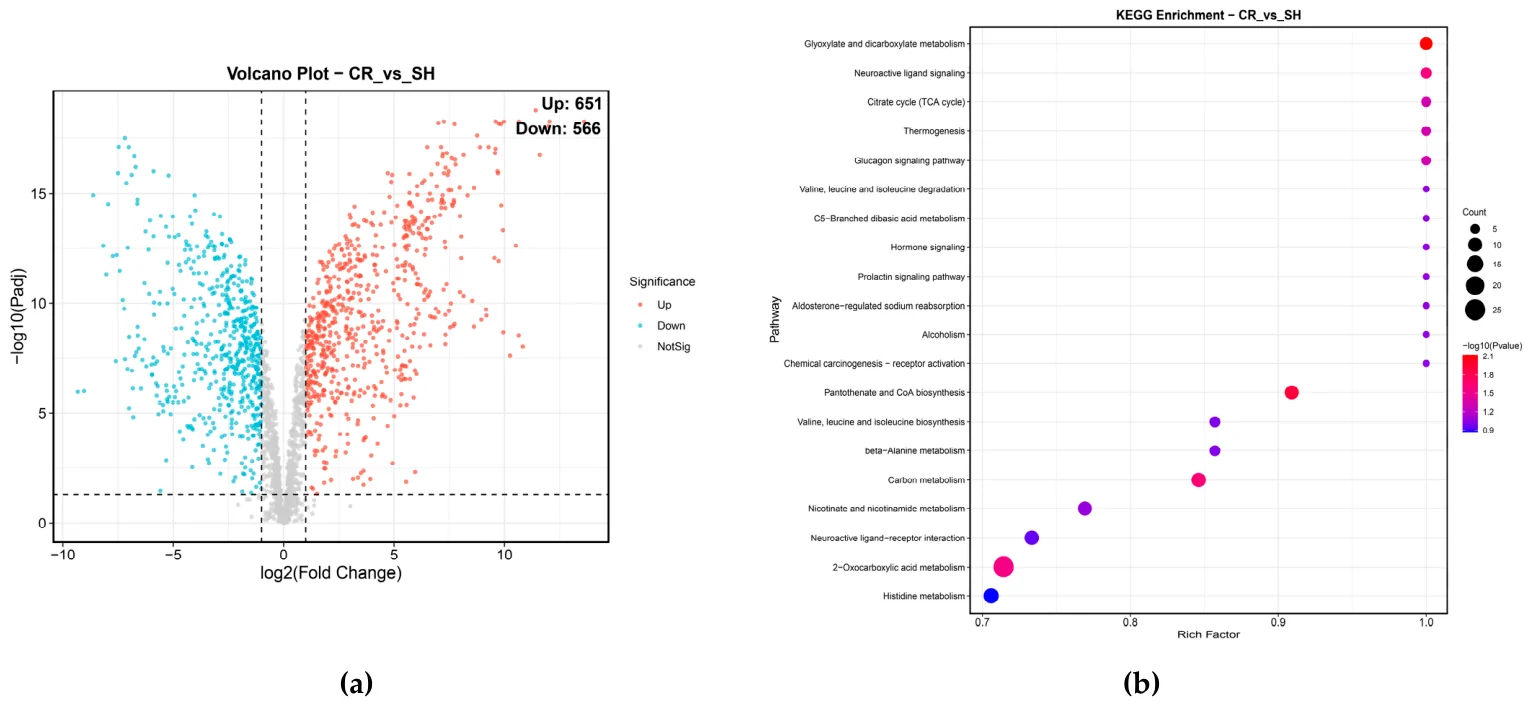

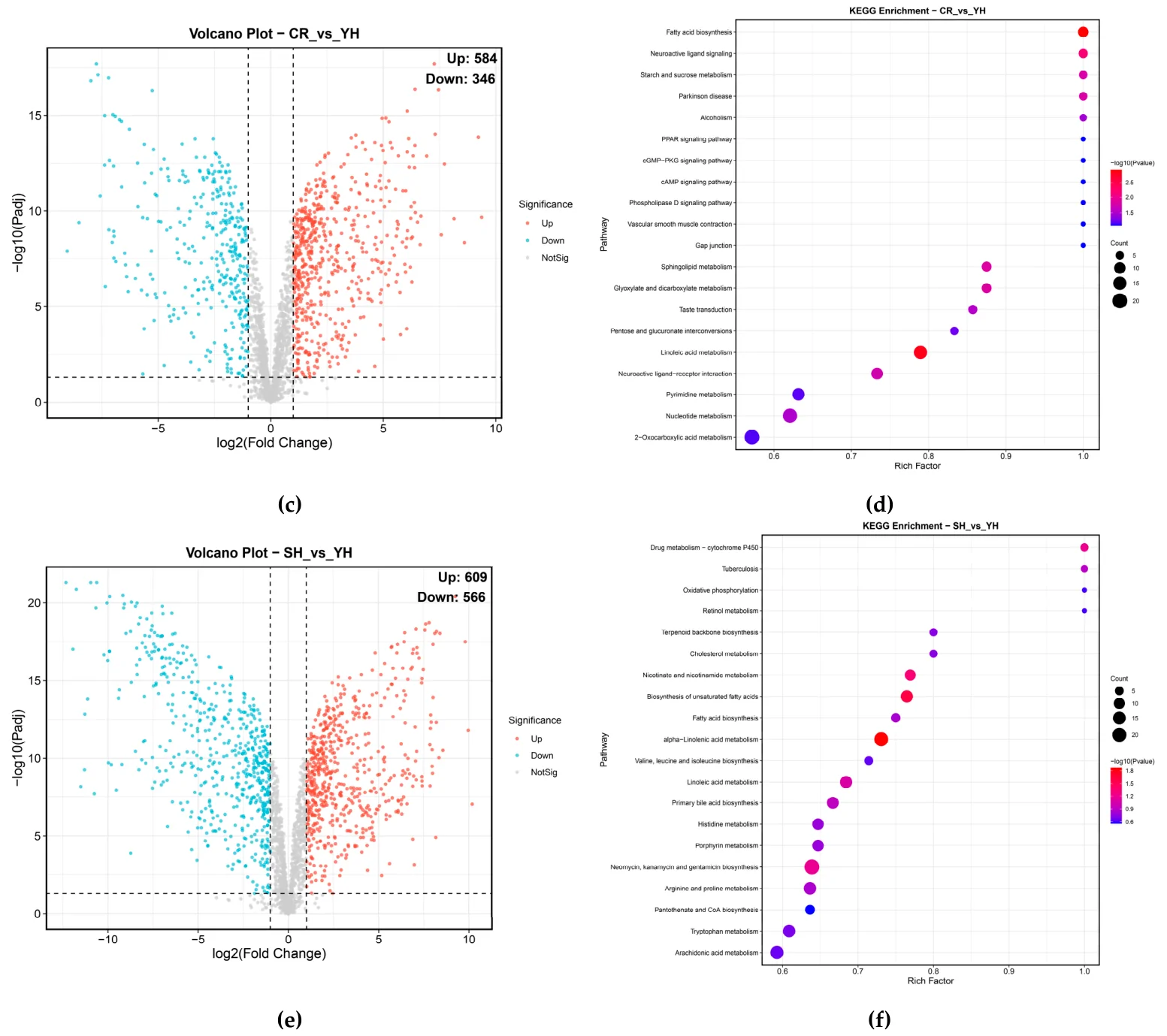
Volcano Plots and Enrichment Analysis of Differential Metabolites Across Three Cattle Breeds. (**a**) Differential metabolites between CR and SH. (**b**) Differential pathway analysis between CR and SH. (**c**) Differential metabolites between CR and YH. (**d**) Differential metabolites between CR and YH. (**e**) Differential metabolites between SH and YH. (**f**) Differential pathway analysis between SH and YH.

In addition, pairwise comparative analyses of differential metabolites were performed between different cattle groups. A total of 1217 differentially expressed metabolites (DEMs) were screened between the CR and SH groups (VIP ≥ 1, adjusted *p* < 0.05), consisting of 651 upregulated metabolites and 566 downregulated metabolites (Figure 7a). KEGG enrichment showed that these differential metabolites were significantly enriched in pathways involved in carbon energy metabolism, branched-chain amino acid metabolism, hormonal and endocrine signaling, and neural signal transduction (Figure 7b). Only basal fatty acid and polyunsaturated fatty acid metabolic pathways were activated in the SH group. These exhibited strong correlations with physicochemical parameters associated with beef flavor.

A total of 930 differentially expressed metabolites (DEMs) were screened between the CR and YH groups (VIP ≥ 1, adjusted *p* < 0.05), including 584 upregulated metabolites and 346 downregulated metabolites (Figure 7c). These differential metabolites were significantly enriched in pathways related to lipid metabolism, the PPAR signaling pathway, intracellular second messenger signaling pathways, carbon and carbohydrate metabolism, neural signal transduction, and nucleotide metabolism (Figure 7d). The YH group exhibited remarkable activation of lipid metabolic pathways. However, energy allocation in this group was biased toward fat synthesis, with a lack of metabolic pathways that accelerate muscle deposition. Combined with metabolic disorders associated with neural stress, the overall growth performance of the YH group was inferior to that of the CR group.

A total of 1175 differentially expressed metabolites (DEMs) were screened between the SH and YH groups (VIP ≥ 1, adjusted *p* < 0.05), including 609 upregulated metabolites and 566 downregulated metabolites (Figure 7e). The differential metabolites were predominantly enriched in pathways involved in lipid anabolism, branched-chain amino acid biosynthesis, mitochondrial oxidative phosphorylation, vitamin coenzyme metabolism, and antioxidant metabolism (Figure 7f). The YH group showed simultaneous enrichment of lipid synthesis and polyunsaturated fatty acid metabolic pathways (beneficial for meat quality improvement), as well as branched-chain amino acid biosynthesis and oxidative phosphorylation pathways (facilitating growth).

### 3.6. Combined Analysis of Microbiome and Metabolome

**Figure 8:**
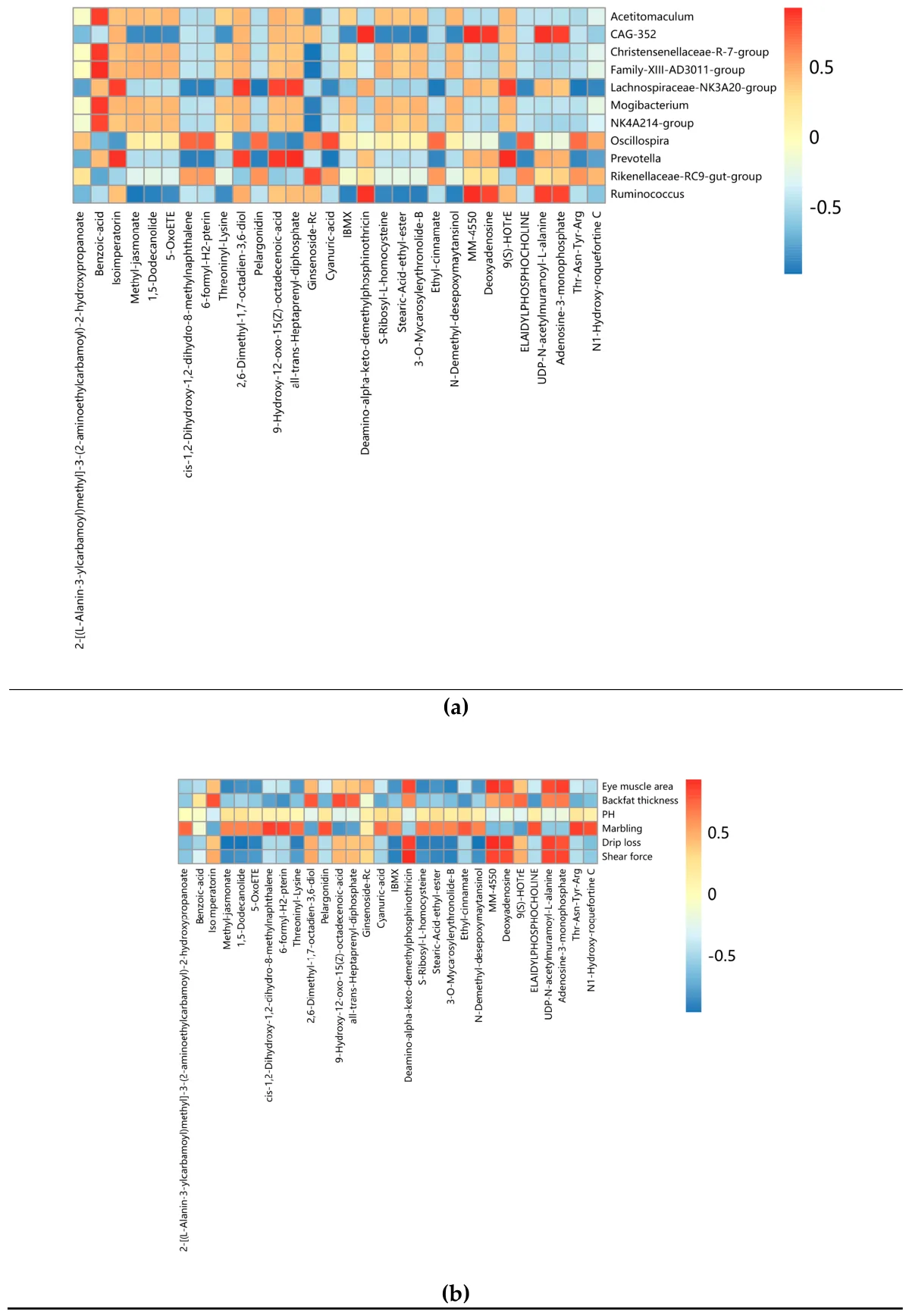
Correlation analysis among rumen microorganisms, metabolites, and phenotypic traits of three cattle breeds. (**a**) Correlation analysis of microbiota and metabolites. (**b**) Correlation analysis of meat quality traits and metabolites.

Since a large number of uncultured and unclassified taxa exist at the species level in the third-generation 16S rRNA species annotation, representative differential microbial taxa at the genus level and differential metabolites were selected for correlation analysis to ensure the reliability of integrated analysis. Subsequently, an interaction heatmap of differential microbial taxa and differential metabolites was constructed (Figure 8).

For metabolite screening, distinct metabolite clusters were identified via LEfSe analysis, and the top 10 group-specific metabolites of each group were selected for subsequent analysis. For microbial screening, differential characteristic taxa were obtained from pairwise comparisons implemented in LEfSe analysis. At the genus taxonomic level, uncultured and unannotated species were excluded before integrated downstream analysis.

Correlation analysis was conducted between microorganisms and metabolites. The results revealed that Acetitomaculum exhibited a strong positive correlation with 2-[(L-Alanin-3-ylcarbamoyl)methyl]-3-(2-aminoethylcarbamoyl)-2-hydroxypropanoate and a strong negative correlation with Ginsenoside Rc. Genus CAG_352 was strongly negatively correlated with nine metabolites and strongly positively correlated with five metabolites. *Christensenellaceae_R_7_group*, Family_XIII_AD3011_group, Mogibacterium, and NK4A214_group all showed strong positive correlations with Benzoic acid and strong negative correlations with Ginsenoside Rc.

Lachnospiraceae_NK3A20_group had strong positive correlations with five metabolites and strong negative correlations with eight metabolites. Oscillospira was strongly positively correlated with 1,5-Dodecanolide and Cyanuric acid, while displaying strong negative correlations with Isoimperatorin, 9-Hydroxy-12-oxo-15(Z)-octadecenoic acid, and all-trans-Heptaprenyl diphosphate. Prevotella showed strong positive correlations with five metabolites and strong negative correlations with seven metabolites. Rikenellaceae_RC9_gut_group was strongly positively correlated with Ginsenoside Rc. *Ruminococcus* had strong positive correlations with five metabolites and strong negative correlations with nine metabolites.

Furthermore, correlation analysis was performed between metabolites and meat quality-related indicators. Eye muscle area was strongly positively correlated with five metabolites and strongly negatively correlated with eight metabolites. Backfat thickness presented strong positive correlations with Isoimperatorin and 9-Hydroxy-12-oxo-15(Z)-octadecenoic acid, as well as strong negative correlations with 6-formyl-H2-pterin, Ethyl cinnamate, and ELAIDYLPHOSPHOCHOLINE. Marbling was strongly positively correlated with six metabolites and strongly negatively correlated with 9-Hydroxy-12-oxo-15(Z)-octadecenoic acid. Drip loss exhibited strong positive correlations with five metabolites and strong negative correlations with nine metabolites. Shear force was strongly positively correlated with five metabolites and strongly negatively correlated with seven metabolites.

## 4. Discussion

Beef meat quality has always been one of the most important traits of great concern to consumers. Therefore, improving beef quality has become a key goal of modern animal husbandry. Intramuscular fat content, muscle fiber content, muscle water holding capacity, and muscle cross-sectional area are all crucial factors affecting meat quality. Meanwhile, these indicators are regulated by various factors including cattle breed, growth environment, and age. With the increase in intramuscular fat content, meat tenderness, flavor, shear force, drip loss rate, and marbling score are all significantly improved [47]. Significant differences were observed in intramuscular fat, meat color, pH value, tenderness, water loss rate, and flavor-related lipid components between Angus cattle and Xinjiang Brown cattle. In addition, their microbial community structures also differed significantly. Certain specific differential intestinal microbiota can regulate lipid metabolism, thereby affecting fat deposition and meat quality traits of beef [48]. This study showed that the hip width of Yanhuang cattle was significantly lower than that of Simmental hybrid cattle and Chinese steppe red cattle, while no significant differences were observed in body length, body height, chest girth and abdominal girth among the three breeds. The eye muscle area, backfat thickness, drip loss rate, and shear force of Simmental hybrid cattle were significantly higher than those of Chinese steppe red cattle and Yanhuang cattle, whereas the marbling score of Simmental hybrid cattle was significantly lower than that of the other two breeds. These results indicated that the meat quality of Yanhuang cattle and Chinese steppe red cattle was superior to that of Simmental hybrid cattle.

These findings support the view that Yanhuang cattle and Chinese steppe red cattle possess relatively better meat quality, and provide a comprehensive phenotypic basis for understanding the differences in growth performance and meat quality among the three cattle breeds.

In addition, studies have demonstrated that the composition of fatty acids and amino acids affects the flavor, juiciness, and nutritional value of meat. A study on the meat quality of Qinchuan cattle and Nanyang cattle finally identified L-serine as a key functional metabolite promoting intramuscular fat deposition [49]. Other studies have conducted integrated transcriptomic and metabolomic analyses to compare molecular discrepancies in beef cattle with varying intramuscular fat levels, identifying four core pathways, such as fatty acid metabolism and unsaturated fatty acid biosynthesis, that modulate intramuscular fat deposition in beef cattle. WGCNA analysis was utilized in these studies to screen ITGB1 as the hub gene responsible for regulating intramuscular fat content [50]. In addition, previous studies have demonstrated discrepancies in rumen microbiome and metabolome profiles between yaks and Simmental cattle across different lactation stages, with particularly prominent differences in the contents of amino acids and fatty acids. Yaks harbor unique microbial communities, and the pathways associated with amino acid biosynthesis and fatty acid metabolism are markedly upregulated. This fundamentally elucidates the intrinsic mechanism behind the superior amino acid and fatty acid composition, as well as the overall higher nutritional value of yak milk compared with Simmental milk [51].

Leucine, isoleucine, and lysine determine muscle growth, tenderness, and nutritional quality of beef [52]. Furthermore, lysine is an essential limiting amino acid, and lysine supplementation can effectively improve meat quality and flavor [53]. In the present study, the contents of Asp, Arg, Phe, Ile, Leu, and Lys in Simmental hybrid cattle were significantly higher than those in Yanhuang cattle and Chinese steppe red cattle. The levels of His, Gly, Ala, Val, and Pro in Simmental hybrid cattle and Chinese steppe red cattle were markedly higher than those in Yanhuang cattle, while the Thr and Met contents of Chinese steppe red cattle were significantly greater than those of Simmental hybrid cattle and Yanhuang cattle. Unsaturated fatty acids (UFAs) are the primary fatty acids affecting beef flavor, among which polyunsaturated fatty acids (PUFAs) account for the largest proportion [54]. The contents of MA, PA, POA, SA, OA, LA, and ALA in Chinese steppe red cattle and Yanhuang cattle were significantly higher than those in Simmental hybrid cattle, and the abundance of PUFAs in Yanhuang cattle and Chinese steppe red cattle was also higher than that in Simmental hybrid cattle. Therefore, Chinese steppe red cattle and Yanhuang cattle are expected to be optimal choices for high-quality beef production and possess great market development potential. Numerous studies have demonstrated that the gut microbiome is closely associated with host amino acid and lipid metabolism, thereby regulating meat quality traits related to muscle fiber cross-sectional area, drip loss, and meat tenderness [55,56]. Diet, genetic background, age, sex, and intestinal environment of different hosts shape the structure, abundance, and function of microbial communities and alter microbial metabolic patterns [57,58].

Significant differences in meat flavor compounds, colonic microbiota, and metabolites were observed in the longissimus dorsi muscle of Duroc × Landrace × Yorkshire (DLY) pigs with high and low intramuscular fat (IMF) content. In the colon of high-IMF pigs, *Dorea* was significantly enriched and positively correlated with major volatile organic compounds (VOCs), while *Treponema*, *Alloprevotella*, and *Cryptobacteroides* were significantly decreased. Ester compounds were predominantly enriched in high-IMF pork, and differential metabolites were mainly enriched in nucleotide metabolism and pyrimidine metabolism pathways [59].

In addition, a study took Jiaxian red cattle as experimental animals and adopted multi-omics technologies including rumen metagenomics, rumen fluid metabolomics, muscle metabolomics, and muscle transcriptomics. By comparing beef cattle samples with divergent intramuscular fat contents, four core rumen microorganisms, namely Cnuella, Gaetbulibacter, Moheibacter, and Lacibacter, were screened out. It was verified that these microbial communities could enrich α-linolenic acid, a key intermediate metabolite in the rumen [60]. Integrated analysis of host microbiome and metabolome indicated that rumen microbiota are closely associated with lipid, carbohydrate, and protein metabolism. Meanwhile, meat quality and animal growth traits are strongly correlated with alterations in gut microbial communities involved in lipid metabolism [57,61]. In the present study, microbial diversity analysis was performed. The alpha diversity results showed that the gut microbial richness and diversity of Chinese steppe red cattle and Yanhuang cattle were higher than those of Simmental hybrid cattle. Firmicutes was the most dominant bacterial phylum among the three cattle breeds. Furthermore, LEfSe analysis revealed that the rumen microbiota of Simmental cattle possessed richer differential microbial taxa.

Metabolites, as the end products of gene function execution and direct reflections of cellular physiological status, can provide insights that are more closely related to phenotypic characteristics [62,63]. In this study, a total of 2425 metabolites were identified and annotated. Metabolites unique to each group were screened via LEfSe analysis. Pathway enrichment analysis revealed that pathways including branched-chain amino acid degradation, tricarboxylic acid cycle, carbon metabolism, biosynthesis of amino acids, nutrient absorption and transport, as well as lipid metabolism pathways (linoleic acid metabolism and steroid biosynthesis) were significantly enriched in the CR group. The activation of these pathways involved in energy metabolism, amino acid biosynthesis, and nutrient uptake suggests enhanced nutrient absorption and energy turnover in beef cattle of the CR group, which facilitates muscle protein deposition and thereby improves growth rate and feed utilization efficiency. In addition, the enriched lipid metabolism pathways indicate altered lipid metabolism status, which may participate in regulating beef quality traits.

KEGG enrichment analysis revealed that differential metabolites were mainly involved in fatty acid biosynthesis, polyunsaturated fatty acid metabolism, amino acid degradation, carbohydrate metabolism, and purine nucleotide metabolism pathways, among which the enrichment of lipid biosynthesis and polyunsaturated fatty acid metabolism pathways was particularly prominent. These results suggest that beef cattle in the SH group retain the metabolic capacity for fat deposition and the production of flavor precursors. Meanwhile, activated amino acid degradation pathways and altered energy metabolism characteristics imply suboptimal protein turnover and energy utilization efficiency in this group, which may underlie the inferior growth performance, body weight gain, and lean meat deposition observed in SH cattle. KEGG enrichment analysis of metabolites specifically enriched in the YH group revealed significant enrichment in intracellular signaling pathways such as cAMP, cGMP-PKG, Ras, Rap1, and calcium signaling pathways, as well as pathways related to hormone secretion, lipolysis, vascular smooth muscle contraction, neural regulation, and ferroptosis. These results suggest that beef cattle in the YH group may regulate systemic lipid metabolism via hormone-related signal transduction, facilitating intramuscular fat deposition and further improving beef tenderness and flavor. Moreover, the enriched pathways associated with vascular smooth muscle contraction and cell communication may participate in the homeostasis regulation of tissue blood circulation, promote the transport of nutrients to muscle and adipose tissues, and potentially support both animal growth and meat quality formation. Previous studies on indigenous Portuguese cattle breeds reported that the liver content of alpha-linolenic acid and the activity of related metabolic pathways in Barrosã cattle were significantly higher than those in Alentejana cattle [64]. In addition, a comparative study on Jiaxian red cattle (a native Chinese yellow cattle breed), Angus cattle, and Simmental cattle revealed that the alpha-linolenic acid metabolism pathway was significantly enriched in both the longissimus dorsi muscle and rumen contents, and this pathway was markedly upregulated in Jiaxian red cattle [60]. This indicates that lipid metabolism pathways play a crucial role in the formation of meat quality. Correlation analysis between microbiota and metabolites showed that Acetitomaculum exhibited a strong positive correlation with Benzoic acid and a strong negative correlation with Ginsenoside Rc. Genus CAG_352 was strongly negatively correlated with nine metabolites and strongly positively correlated with five metabolites. *Christensenellaceae_R_7_group*, Family_XIII_AD3011_group, Mogibacterium, and NK4A214_group all showed strong positive correlations with Benzoic acid and strong negative correlations with Ginsenoside Rc.

There are several limitations to this study. First, all the results were derived from correlation analyses, and no functional verification experiments such as microbial functional sequencing, in vitro fermentation, or strain supplementation were conducted to confirm the causal relationships among microorganisms, metabolites, and meat quality indicators, resulting in an insufficiently in-depth interpretation of flavor formation pathways. Second, cross-sectional static sampling was adopted in this experiment without time-series dynamic samples, which made it impossible to elucidate the dynamic regulatory process of flavor substance accumulation throughout the growth cycle.

In addition, each group included only six experimental animals, representing a limited sample size. Such a small sample size may compromise the statistical power of multivariate analyses of microbiome and metabolome data and correlation analysis, and limit the generalizability of our results. Therefore, the conclusions drawn in this study require further verification with larger sample cohorts. Follow-up studies should increase the sample size to enhance statistical power and obtain more stable and robust experimental outcomes.

## 5. Conclusions

Simmental hybrid cattle showed better performance in some growth-related traits and amino acid contents. In contrast, Yanhuang cattle and Chinese steppe red cattle had better meat quality, with higher marbling scores, lower drip loss and shear force, and higher levels of unsaturated fatty acids. Microbial analysis showed that Yanhuang cattle and Chinese steppe red cattle had greater rumen microbial richness and diversity, with distinct microbial community compositions among breeds. Metabolomic analysis indicated that breed-related metabolic differences were mainly involved in lipid metabolism, amino acid metabolism, carbohydrate metabolism, and energy metabolism pathways. Correlation analysis between microbes and metabolites suggested that specific rumen microorganisms may regulate metabolic processes and influence changes in meat quality-related metabolites. In conclusion, the present study revealed that the differences in growth performance and meat quality among Chinese steppe red cattle, Simmental hybrid cattle, and Yanhuang cattle may be attributed to the reprogramming of amino acid metabolism and the deposition process of lipid substances. These findings also provide valuable clues for further identification of candidate genes and metabolites associated with meat quality traits.

## Figures and Tables

**Table 1:** Dietary ingredients and nutritional levels of diets (air/dry matter basis, %).

Ingredient	Proportion (%)	Nutrient Levels	Content (%)
Leymus chinensis	60.00	DM	89.14
Corn	25.00	CP	15.63
Soybean meal	8.00	EE	3.72
Peanue meal	4.00	NDF	57.65
Sodium bicarbonate	0.70	AGF	34.74
Limestone	0.30		
Premix	2.00		
Total	100.00		

**Table 2:** Analysis of Growth and Meat Quality Traits in CR cattle, SH cattle, and YH cattle (*n* = 6 in each breed).

Items	CR	SH	YH	SEM	*p*-Value
Comparison of growth					
Body length (cm)	126.67	131.50	128.50	2.056	0.275
Body height (cm)	114.83 ^b^	120.50 ^a^	120.67 ^b^	1.389	0.014
Hip cross height (cm)	118.67 ^b^	125.83 ^a^	123.68 ^a^	1.289	<0.01
Chest girth (cm)	169.33	171.333	173.00	1.923	0.423
Abdominal girth (cm)	184.67	189.500	189.00	2.626	0.383
Comparison of meat quality					
Live weight (kg)	413.33	416.34	410.0	8.014	0.857
Carcass weight (kg)	240.50	228.01	236.67	9.787	0.659
Net meat weight (kg)	198.00	196.51	196.18	6.072	0.975
Bone weight (kg)	41.14	42.03	41.67	0.826	0.752
Dressing percentage (%)	58.17	54.88	57.67	2.091	0.504
Net meat percentage (%)	47.82	47.16	47.84	0.737	0.761
Comparison of meat quality					
Eye muscle area (cm^2^)	103.90	127.72 ^a^	118.38 ^b^	1.801	<0.01
Backfat thickness (cm)	0.94 ^b^	1.22 ^a^	0.84 ^b^	0.037	<0.01
pH	6.16	5.87	6.13	0.173	0.443
Marbling	3.91 ^a^	2.16 ^b^	4.08 ^a^	0.093	<0.01
Drip loss (%)	3.35 ^c^	8.71 ^a^	6.84 ^b^	0.201	<0.01
Shear force (kg·cm^2^)	3.67 ^c^	8.58 ^a^	4.39 ^b^	0.213	<0.01
Comparison of 16 amino acid composition (g/100 g)					
Asp	1.86 ^c^	2.18 ^a^	2.02 ^b^	0.045	<0.01
Glu	3.54 ^ab^	3.69 ^ab^	3.24 ^b^	0.110	0.035
Ser	1.19 ^a^	0.88 ^ab^	0.63 ^b^	0.133	0.029
His	1.01 ^a^	1.10 ^a^	0.87 ^b^	0.029	<0.01
Gly	1.02 ^a^	1.04 ^a^	0.86 ^b^	0.037	<0.01
Thr	0.99 ^b^	1.02 ^b^	1.15 ^a^	0.022	<0.01
Arg	1.37 ^b^	1.49 ^a^	1.41 ^b^	0.022	<0.01
Ala	1.31 ^a^	1.28 ^a^	1.22 ^b^	0.018	0.01
Tyr	0.88	0.89	0.84	0.032	0.507
Val	1.13 ^b^	1.23 ^a^	1.04 ^c^	0.026	<0.01
Met	0.54 ^b^	0.61 ^b^	0.72 ^c^	0.034	<0.01
Phe	0.89 ^c^	1.21 ^a^	1.06 ^b^	0.026	<0.01
Ile	1.02 ^b^	1.19 ^a^	1.03 ^b^	0.039	0.013
Leu	1.55 ^c^	1.90 ^b^	1.78 ^a^	0.029	<0.01
Pro	0.82 ^a^	0.83 ^a^	0.73 ^b^	0.026	0.038
Lys	1.72 ^b^	2.13 ^a^	1.76 ^b^	0.018	<0.01
Comparison of 7 Fatty acids contents (g/100 g)					
MA	2.56 ^b^	0.38 ^c^	3.29 ^a^	0.149	<0.01
PA	25.69 ^a^	8.81 ^b^	28.703 ^a^	2.928	<0.01
POA	5.62 ^a^	0.62 ^b^	5.16 ^a^	0.213	<0.01
SA	14.44 ^a^	2.55 ^b^	14.42 ^a^	0.398	<0.01
OA	44.93 ^a^	13.73 ^b^	46.18 ^a^	3.539	<0.01
LA	6.18 ^a^	1.58 ^c^	3.52 ^b^	0.232	<0.01
ALA	0.60 ^a^	0.09 ^c^	0.26 ^b^	0.022	<0.01

^a–c^ Different superscripts within a row indicate significantly different values for *p*-value < 0.05.

## Data Availability

The original contributions presented in this study are included in the article/Supplementary Material. Further inquiries can be directed to the corresponding author.

## Supplementary Materials

The following supporting information can be downloaded at: https://www.mdpi.com/article/10.3390/ani16162577/s1, Table S1: Differential Metabolites Identified among the CR, SH, and YH Groups.
